# Metaproteomic and 16S rRNA Gene Sequencing Analysis of the Infant Fecal Microbiome

**DOI:** 10.3390/ijms20061430

**Published:** 2019-03-21

**Authors:** Laetitia Cortes, Harm Wopereis, Aude Tartiere, Julie Piquenot, Joost W. Gouw, Sebastian Tims, Jan Knol, Daniel Chelsky

**Affiliations:** 1 Caprion Biosciences, 201 President Kennedy, Suite PK-3900, Montreal, QC H2X3Y7, Canada; lcortes@caprion.com (L.C.); aude.tartiere@gmail.com (A.T.); juliepiq@gmail.com (J.P.); 2 Danone Nutricia Research, Gut Biology and Microbiology, Uppsalalaan 12, 3584 CT Utrecht, The Netherlands; harm.wopereis@wur.nl (H.W.); joost.gouw@danone.com (J.W.G.); sebastian.tims@danone.com (S.T.); jan.knol@wur.nl (J.K.); 3 Laboratory of Microbiology, Wageningen University, 6708 PB Wageningen, The Netherlands

**Keywords:** metacluster, metabolism, infants, mass spectrometry, fecal, intestinal, microbiome

## Abstract

A metaproteomic analysis was conducted on the fecal microbiome of eight infants to characterize global protein and pathway expression. Although mass spectrometry-based proteomics is now a routine tool, analysis of the microbiome presents specific technical challenges, including the complexity and dynamic range of member taxa, the need for well-annotated metagenomic databases, and high inter-protein sequence redundancy and similarity. In this study, an approach was developed for assessment of biological phenotype and metabolic status, as a functional complement to DNA sequence analysis. Fecal samples were prepared and analysed by tandem mass spectrometry and a homology-based meta-clustering strategy was used to combine peptides from multiple species into representative proteins. In total, 15,250 unique peptides were sequenced and assigned to 2154 metaclusters, which were then assigned to pathways and functional groups. Differences were noted in several pathways, consistent with the dominant genera observed in different subjects. Although this study was not powered to draw conclusions from the comparisons, the results obtained demonstrate the applicability of this approach and provide the methods needed for performing semi-quantitative comparisons of human fecal microbiome composition, physiology and metabolism, as well as a more detailed assessment of microbial composition in comparison to 16S rRNA gene sequencing.

## 1. Introduction

The infant gut microbiome has been the subject of considerable attention, as its early development is thought to be critically important for a healthy immune maturation [1,2]. Therefore, several studies have attempted to understand the impact of antibiotics [3,4,5,6], Caesarean-section (C-section) [7,8], breast and formula feeding [5,9,10,11] and age [12,13], particularly on bacterial taxonomic composition.

The infant microbiome shows dynamic changes from birth until around three years of age, when it approximates that of the adult [2,12]. After oxygen in the gut is consumed by pioneering microbes in the first days of life, anaerobic species of *Bifidobacterium* and *Bacteroides* typically dominate in breastfed, vaginally born healthy infants [1,2,9,10,11,12]. In these infants, the early colonizers have been shown to be vertically transmitted from the mother [9,12]. C-section delivery, which limits normal vertical transfer during birth, can result in several months delay in this profile, which is temporarily substituted by higher levels of facultative anaerobes and skin microbes, such as *Propionibacterium* and *Staphylococcus* [7,11,14]. Antibiotic treatment can reduce phylogenetic diversity, leading to displacement with potential pathogens and a delay in microbiota and immune maturation [6]. The main driving factor of the maturation of the gut microbiome is early life nutrition. Human milk contains complex carbohydrates, known as human milk oligosaccharides (HMOs). These compounds are mainly consumed by *Bifidobacterium* species, and have sustained effects on gut microbiome composition, even after introduction of solids to the infant-diet [12,15].

Most studies of the infant microbiome have been based on culture enumeration, quantitative PCR and fluorescence in situ hybridization. More recently, high throughput DNA sequencing methods have provided a more detailed view of gut microbial complexity, however with limited insights on its functionality [10,12,16]. Of the high-throughput sequencing methods, 16S rRNA gene sequencing has become widely popular because of its relative low cost. However, the level of detail provided by this method is prone to variability due to amplification biases [17,18] and suffers from poor resolution at the species or strain level [19,20]. Generating this more detailed information is possible with whole genome shotgun sequencing (metagenomics) [21], but this approach is expensive, requires substantial data analysis [22] and suffers from a lack of standardization, which introduces biases [21,23].

Proteomics has recently emerged as an alternative to the genomics approach as it bypasses all issues related to amplification, a major component of the variability identified in 16S rRNA gene or metagenomics approaches [24]. This approach has proven to be useful in the study of microbial communities, bringing insights on taxonomies present as well as detection of key enzymes in, for example, activated sludges, acid mine drainage biofilms and marine systems [25]. Due to the diversity of bacterial species and therefore the proteins detected, the most useful approach has been to treat the proteome as representing a single community, or metaproteome [26,27,28]. By combining proteins of like function and with high homology, a picture can be obtained for the proteomic state of the microbiome as a whole [28,29,30]. Since many of these proteins are involved in various enzymatic pathways, the metabolic state of the microbiome can be derived and measured in response to various conditions and perturbations.

Recently, a number of studies have applied metaproteomics to characterize the infant gut in order to better understand the implications of the observed taxonomic differences under different conditions, ages and treatments [31,32,33,34,35]. In order to further develop and expand this approach, we analysed fecal samples from multiple infants, each representing factors known to influence gut microbial development; including one pair of twins, an infant treated with antibiotics, one born by C-section delivery, and incorporating diets of human milk, infant formula or a combination of the two (see Section 4). Data were evaluated in terms of taxonomic detail, based on individual peptide sequences. Peptides were then associated into protein metaclusters to assess the community-wide metabolic status of each infant.

## 2. Results

Metaproteomics analysis, based on shotgun sequencing of the enriched bacterial pellets, resulted in the identification of approximately 3500 unique bacterial peptide sequences in each sample and 14,216 overall (Table 1). These identifications were based on 40,924 independent spectra. By comparison, the number of host peptide spectra detected represents 18% of the total, which is the best estimate of the relative proportion of host protein in the samples.

**Figure 1:**
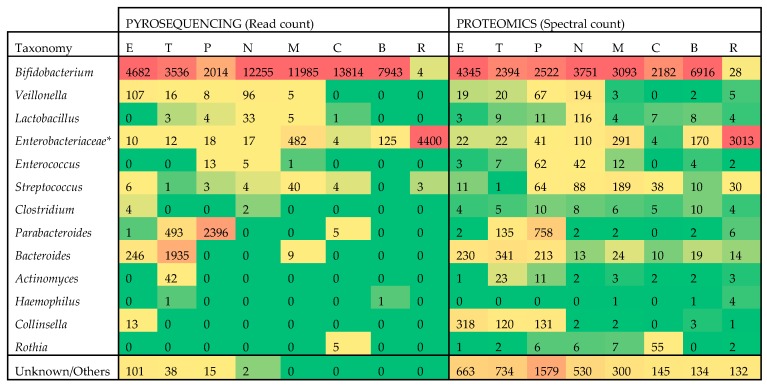
Heat map comparison of pyrosequencing and metaproteomics results across the most abundant taxa. Data is summarized at the genus level, except for the family *Enterobacteriaceae* which incorporates multiple genera to simplify comparisons. Values are colored in shades of green to yellow to red, indicating low, medium and high abundance, respectively. Actual read counts and spectral counts are also provided. Subjects are identified by a letter code here and in other figures and tables (E, T, P, N, M, C, B, R).

Using the identified bacterial peptides, insights can be gained into the bacterial genera/species distribution as well as the functionality of the microbiome. The former was addressed first and a total of 253 different bacterial species could be detected by interrogating the proteomics data (Supplemental Tables S1 and S2). To evaluate these results, a more traditional technique, pyrosequencing, was applied to the same samples which led to the identification of 30 different species (Supplemental Table S3). A comparison was made between 16S rRNA gene sequencing and proteomics in terms of the commonly identified bacterial taxa and their relative abundance. The limited resolution of 16S rRNA analysis is well known [19,20], but the more abundant genera were readily detected by both methods, which allowed for a comparison of measured rank order and/or abundance. In Figure 1, the top 12 bacterial genera and one bacterial family were compared with a heat map.

Multiple genera (*Enterobacter*/*Escherichia*/*Shigella* for pyrosequencing and *Klebsiella*/*Escherichia*/*Citrobacter* and 11 other genera for proteomics) were combined under their family *Enterobacteriaceae*, to simplify comparison, since these genera did not match well between methods, potentially due to the high level of homology of 16S rRNA gene sequences within this family. Read counts were used as the metric for pyrosequencing and spectral counts for proteomics. The scale of the two measures was similar and the general distribution of the genera was comparable. For example, subject R stood out as it was nearly completely lacking, by both measures, the genus *Bifidobacterium* which was replaced by *Enterobacteriaceae* in this antibiotic-treated subject. Similarly, the genus *Parabacteroides* was on par with *Bifidobacterium* in subject P (C-section born, and vaccinated just prior to fecal sample collection) while *Bacteroides* showed elevated abundance in subject T. Both methods mostly agreed in their profile of the monozygotic twins, N and M, including the observation that subject M had relatively lower levels of *Veillonella* and *Lactobacillus* than subject N. The Spearman rank order correlation between the two datasets is 0.719 while the Pearson correlation is 0.795, further demonstrating a close match in relative quantitation of taxonomic composition by the two methods.

Although the two methods were consistent in taxonomic profiling, there were many more cases of low representation or missing data in the pyrosequencing data. Furthermore, these 12 genera and one family accounted for most of the pyrosequencing data, while the proteomic data identified many other genera (see “unknown/others” in Figure 1). In total, 65 additional genera were identified only through proteomics (Supplemental Table S2).

**Figure 2:**
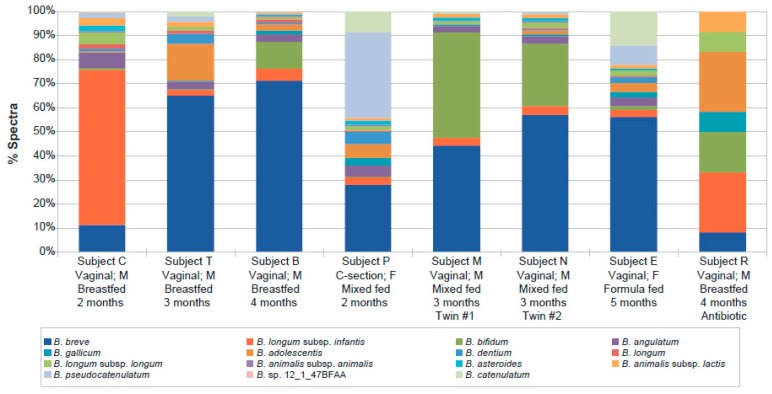
Relative *Bifidobacterium* species abundance per infant as detected by metaproteomics. Strain-level assignments were combined at the species level by summing spectral counts identified in individual strains.

The composition of each individual microbiome was clearly unique at the genus level. While it is difficult to resolve below this level with pyrosequencing, the metaproteomics data could resolve at the species and even strain level (Supplemental Table S4). Since *Bifidobacterium* was the dominant genus in all but one subject, the species distribution within that genus was examined in each subject (Figure 2). A high degree of heterogeneity was observed, although the twins had, by far, the most similar profiles. The dominant species in most subjects was *B. breve,* which increased in representation with age. *B. bifidum* was the second most common species in the monozygotic twins, while *B. longum* subsp. *infantis* was dominant in one of the youngest subjects (C), and *B. pseudocatenulatum* was most common in subject P, the youngest subject analysed and born by C-section.

**Figure 3:**
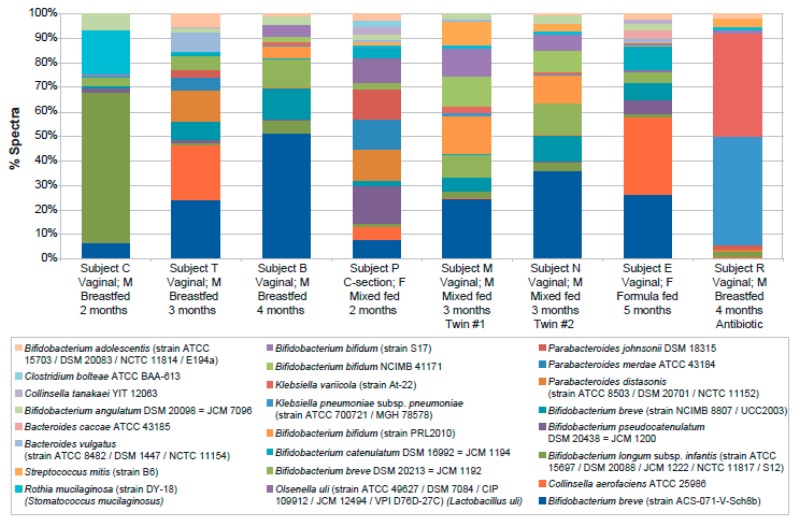
Relative strain-level abundance per infant as detected by metaproteomics. Included are the 24 strains represented by at least 20 spectra in at least one infant.

In Table 2, the number of bacterial strains identified by one or more unique spectra for each subject is provided. If relying on as little as one spectrum or peptide, between 45 and 99 strains could be detected in each subject. If five spectra per strain was used as a threshold for higher confidence, between 9 and 33 strains could be detected in each infant, although the absolute numbers detected can also be affected by the amount of sample analyzed which can differ slightly between subjects. Patterns of relative strain abundance were similar to those observed at the genus level, with *Bifidobacterium* representing the largest group in most infants, twins presenting similar distributions and the infant treated with antibiotics having an altered distribution (Figure 3).

In addition to taxonomic classification, the functionality of the microbiome is another important aspect that can be investigated by proteomics. As outlined above, due to the high complexity of these samples and the different genera, species and strains detected in each subject, comparison of the same sequence across subjects would not be productive. Instead, peptides representing the equivalent protein in each species were clustered together, based on sharing the same gene name and/or peptide evidence and/or the same Uniref50 identifier. The resulting protein metaclusters allowed each microbiome to be treated as a set of common functional features, providing higher power to the analysis. As a result, changes in enzyme pathway representation could be more readily detected. In this study, the total number of microbial metaclusters detected was 1922 (Table 1). An additional 212 host metaclusters were found, representing 11% of the total.

Of the bacterial metaclusters, only 1033 (54%) were associated with a useful annotation. However, when a subset of the remaining individual metaclusters was verified manually, they were all successfully annotated using other databases (e.g., RefSeq, KEGG Pathway, etc.), and mapped to genes included in other metaclusters, suggesting that most or all of the metaclusters could likely be annotated successfully and further combined. Improvements to the metaclustering algorithm may be achieved by either using a different homology database (e.g., COG) or combining multiple databases to provide successful annotation and better clustering potential.

**Figure 4:**
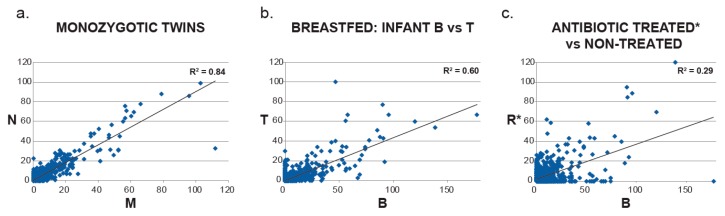
Pairwise correlations of protein metacluster spectral counts of individual infants comparing monozygotic twins (**a**), unrelated breastfed male infants (**b**) and antibiotic-treated vs non-treated infants (**c**). Pearson correlation coefficient values are provided for each comparison. * Antibiotic used was amoxicillin.

In order to compare the metaproteome of each subject, pairwise scatter plots were generated, based on the number of detected spectral counts in each metacluster (Figure 4). As expected, there was a high correlation between the two monozygotic twins (R^2^ = 0.84). Two unrelated males, both breastfed, were highly similar but less so than the twins (R^2^ = 0.60). However, when the antibiotic-treated subject was compared to an untreated subject, the correlation was low (R^2^ = 0.29).

**Figure 5:**
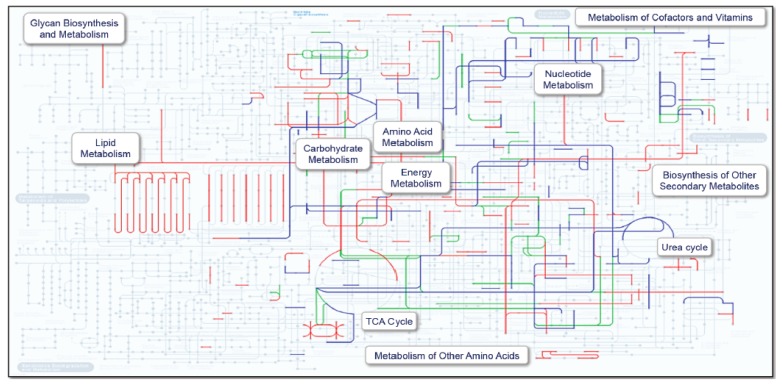
KEGG Pathway analysis using protein metaclusters and corresponding spectral counts. Pathways highlighted in red are more abundant in the antibiotic treated subject. Those in blue are less abundant while green indicates equivalence.

**Figure 6:**
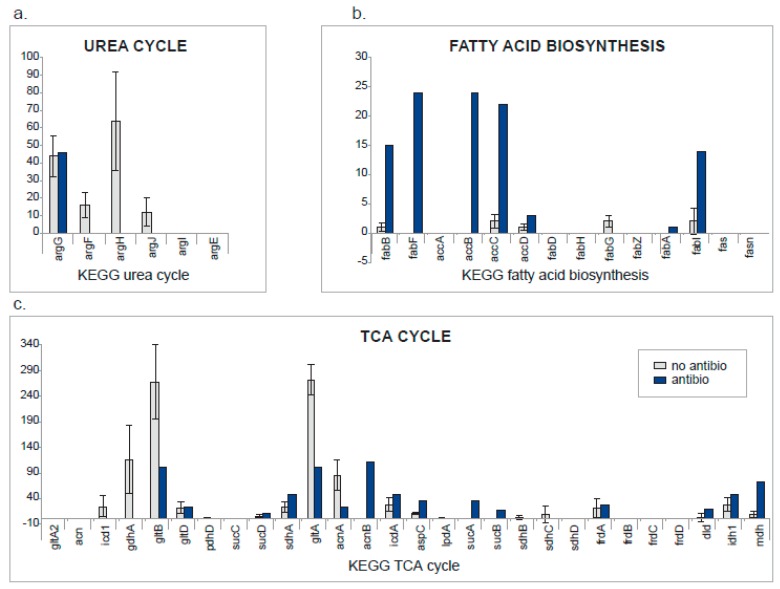
Comparison at the pathway level between infants with and without antibiotic treatment. For the three KEGG pathways showing differences between the two groups (**a**) urea cycle, (**b**) fatty acid biosynthesis and (**c**) TCA (tricarboxylic acid) cycle (a.k.a citric acid or Krebs cycle), the number of spectral counts for all proteins was calculated. For the infant treated with antibiotics, spectral counts detected for each protein are shown (blue bars). For the other infants, the average number of spectral counts and corresponding standard deviations are shown (grey bars).

Because the antibiotic-treated subject was so different from the seven non-treated subjects in terms of both taxonomy and metacluster spectral counts, we chose this comparison to evaluate a method for comparing metabolic pathway expression. This was accomplished by inputting metaclusters with useful annotation into the KEGG Pathway mapping tool [36,37] (Figure 5). The most affected pathways were subsequently evaluated in more detail (Figure 6).

The urea cycle enzymes were well represented in untreated subjects, but only one enzyme was detected in the antibiotic-treated subject. A very different picture is observed for fatty acid biosynthesis, which was upregulated in the antibiotic-treated subject. A mixed result was obtained for the TCA cycle, which lies at the crossroads of the urea cycle and fatty acid biosynthesis and may reflect the differences in microbial energy metabolism between antibiotic-treated and untreated subjects.

## 3. Discussion

In this study, we evaluated the potential benefit of metaproteomics to the field of human microbiome research. Currently, the majority of microbiome studies are based on high throughput DNA sequencing methods, which have revealed the complex nature of the taxa involved, but with only limited insights on functional activity.

In a direct comparison of the taxonomies detected by each method, the mass spectrometry data essentially replicated the phylogenetic composition at the bacterial genus level, providing a similar but potentially more in-depth taxonomic map of each subject than obtained with 16S rRNA sequencing. *Bifidobacteria* represented the largest group in all infants, except for the infant treated with antibiotics. *Bifidobacteria* are typically abundant in the infant microbiome since they are adapted to use human milk oligosaccharides (HMO) as their source of carbon [38]. In addition, high levels of *Bacteroides*/*Parabacteroides* can be seen to distinguish several of the non-treated subjects. These genera are known to be well adapted to feed on mucosal glycans present in the gut and share pathways for feeding on HMO [39]. With either approach, it was clear that the antibiotic-treated subject had a completely different profile from the other subjects, with the *Enterobacteriaceae* family mostly replacing *Bifidobacterium*. However, proteomics revealed that the antibiotic-induced increase of *Enterobacteriaceae* was more specifically associated with two *Klebsiella* species, which are well-known opportunistic pathogens [40].

The metaproteomic analysis also allowed assessing the species distribution within the genus *Bifidobacterium*, which not only revealed a high degree of heterogeneity within each subject, but also different profiles between subjects. The species *B. breve* was most dominant in the majority of infants, followed by other typical infant-type bifidobacterial species, namely *B. longum* subsp. *infantis*, *B. longum* subsp. *longum* and *B. bifidum*. Although the number of infants was low, the proportion of *B. breve* increased with age, a pattern that was observed previously in exclusively breastfed infants, and in infants receiving a combination of breastfeeding and infant formula [41]. Consistent with previous observations [42], *B. longum* subsp. *infantis* represents a majority only in breastfed younger infants, e.g., due to lacto-*N*-neotetraose, a HMO component, which favors this HMO-adapted species as well as *B. breve* [43,44] over those that use mucus, like *Bacteroides* [39].

Proteomics also provided additional information on lower abundance genera as well as significant information at the species and strain level. A total of 183 different taxa were identified by proteomics with strain-level assignments, with an average of 71 in each subject, representing 147 different species and 76 different genera. Of course, these conclusions rely on the assumption that the database used was sufficiently complete. By comparison, pyrosequencing identified only 13 genera overall, with a range of 2 to 8 per subject and no information at the species or strain level. The additional detail afforded by the metaproteomics analysis should be instrumental to better understanding environmental perturbations such as the effects of disease, antibiotic treatments and diet. While this study was too small to make many generalizations about observed differences, the high degree of similarity of the twins at the strain level and the overall high correlation between proteomics and pyrosequencing at the genus level demonstrate that the data is far from random. Interestingly, the profile of the twins closely matches one other subject (B) but less so the remaining subjects. The antibiotic-treated subject differs greatly from the others, but several other subjects are similarly unique, likely for other reasons, including age, mode of delivery, diet or other factors not accounted for in the available clinical information. In future metaproteomics studies of the microbiome, it appears that performing 16S rRNA pyrosequencing on the same samples would offer little benefit. Only a limited number of organisms were identified by this method, most of which were already part of the Human Microbiome Project Database (26 out of 30), and those not present in the publicly available database only represented <0.002% of the total identified spectra.

In addition to the taxonomy information provided by proteomics, a quantitative picture was generated at the enzyme level, which was used to assess pathway and, therefore, metabolic activity. Due to the diversity of bacteria in the gut, each unique protein had a low level of representation in the metaproteomic data, making it difficult to draw conclusions about the status of a single species. However, by grouping functionally similar proteins from each species, metaclusters were created that provided a means of comparing subjects, treating the microbiome of each as the average of all representative functional features. With this approach, it was possible to make high level (Figure 4) as well as detailed comparisons (Figure 5 and Figure 6).

The highest degree of correlation at the metacluster level, observed for the twins (Figure 4), confirmed the strong influence of host genetics and shared environment on the microbiome [45]. In contrast, the antibiotic-treated subject showed the lowest correlation at the metacluster level in a pairwise comparison to a non-treated subject (Figure 4). To assess the differences in metabolic activity of this infant compared to the others, we assigned the metaclusters to enzyme pathways and functional groups. This pathway analysis revealed that untreated infants showed high levels of urea cycle enzymes (Figure 5 and Figure 6). *Bifidobacterium*, dominant in this group, is well adapted to utilize urea, abundant in milk, and turn it into a source of nitrogen usable for the host [46]. The antibiotic-treated subject was high in fatty acid biosynthesis enzymes, potentially due to the inability of *Klebsiella* to efficiently metabolize milk sugars and therefore relying on milk fat instead. The TCA cycle also showed distinct and consistent differences, with nitrogen metabolism enzymes (gdhA, gltB) upregulated in the untreated subjects and carbon metabolism enzymes (sucA, sucB, sdhA, mdh) upregulated in the treated subject.

Further improvements may yield even more comprehensive results. Many of the lower abundance species and metaclusters were represented by small numbers of spectra, limiting confidence in both identity and quantitation. This is mainly due to limitations of the instrumentation to fully sample all peptide ions with each analysis and can be improved by additional fractionation, repeated analysis of each sample, and/or using even more advanced mass spectrometers. Of the metaclusters identified, many were not associated with a meaningful function when using the UniRef database. Improving the quality of the metacluster functional annotation seems achievable by refining the clustering criteria and/or using databases providing better functional annotation [28,47,48] and could potentially bring a deeper understanding of the various pathways and biological functions involved. Finally, cost and throughput should be noted. Mass spectrometry-based approaches involve more effort and take longer than DNA-based analyses, so there is a trade-off for the information obtained. Fecal samples do require more processing steps than most other proteomics samples and fractionation of the digested proteins was necessary to improve the number of peptides detected, thus also increasing the number of mass spectrometry analyses for each sample. These requirements will certainly impact the size of such studies, but the additional insight obtained from the relative quantitation of hundreds of protein metaclusters in each sample, which cannot be otherwise obtained, will likely offset the effort required.

## 4. Materials and Methods

### 4.1. Sample Collection

Fecal samples were obtained from 8 infants between 2 to 5 months of age and living in the Netherlands. Parents were asked to voluntarily participate and information on gestational age, age at collection, gender, feeding mode (breastfed/formula fed), mode of birth and use of concomitant medication was collected (Table 3). Written informed consent was obtained from all participants. Fecal samples were collected by the parents from diapers into 10 ml stool containers (Greiner Bio-One, Kremsmünster, Austria) and kept at 4 °C. Within one hour after collection, laboratory assistants prepared homogenized stool aliquots (5–10) in 2 mL centrifuge tubes, which were subsequently kept and transported on dry-ice for analysis by either 16S rRNA gene sequencing or proteomics. Upon arrival at the respective laboratories and prior to evaluation, samples were kept at −80 °C.

### 4.2. 16S rRNA Gene Sequencing and Bioinformatics

Fecal aliquots were thawed on ice and 20 to 60 mg of each aliquot was mixed with 450 μL DNA extraction buffer (100 mM Tris-HCl, 40 mM EDTA, pH 9.0) and 50 μL of 10% sodium dodecyl sulfate. Phenol-chloroform extractions combined with mechanical lysis of bacterial cells by bead-beating was performed as described by Matsuki et al. [49] except that extracted DNA was re-suspended in 0.1 mL of TE (10 mM Tris-HCl, 1 mM EDTA, pH 8.0). The V3–V5 regions of the 16S rRNA gene were amplified using forward primer 357F, and a ‘bifidobacteria-optimised’ reverse primer 926Rb [17]. The reverse primers included a 12 base-pair error-correcting Golay barcode. PCR was carried out in quadruplicate as previously described [18]. Replicate amplicons were pooled and purified and pyrosequencing was carried out on a 454 GS FLX (Roche, Branford, CT, USA) following the Roche Amplicon Lib-L protocol. The ‘Quantitative Insights Into Microbial Ecology’ (QIIME) v1.9.0 package was used to analyse sequence data [50]. Sequence alignment was carried out using the SILVA rRNA database (SSU_REF111) [51] as reference. Chimera filtering, clustering at 97% sequence identity into operational taxonomic units, and taxonomic assignment were performed using the USEARCH and UCLUST algorithms [52,53]. Rarefaction was performed and singletons were removed. The resulting taxonomic compositions (read counts and relative abundances) were summarized at the genus level.

### 4.3. Proteomics Sample Preparation and Analysis

Each 0.5 gram of test sample was resuspended in 50 mM sodium phosphate buffer (pH8), 0.01% acid-labile surfactant (RapiGest SF Surfactant, Waters, Milford, MA, USA) and placed on a horizontal platform shaker for 10 min at 100 oscillations per min at 20 °C. Remaining large particulate was removed from the suspension with 4 cycles of low-speed centrifugation at 200× *g* for 15 min. After each cycle, the supernatant was collected and kept at 4 °C. At the end, the pooled supernatants were centrifuged at 16,000× *g* for 15 min at 20 °C to collect bacteria. The bacterial pellets were then resuspended in 25 mM Tris-HCl, 150 mM NaCl, 6 M guanidine hydrochloride, pH 7.6 + 0.01% RapiGest and sonicated for 10 min, pulse ON for 30 s and OFF for 10 s. After denaturation, samples were reduced with 5mM *tris*(2-carboxyethyl)phosphine (Thermo Fisher, Waltham, MA, USA), diluted to decrease the guanidine hydrochloride concentration to 1M, and the protein concentration determined by a Bicinchoninic acid assay (Pierce, Waltham, MA, USA). Based on the determined concentration, trypsin was added overnight, followed by a second addition and a 4 h incubation, both at 37 °C (1:50 (*w*:*w*) enzyme:protein ratio each addition, Promega, Madison, WI, USA). Samples were acidified with 0.1% trifluoroacetic acid (Sigma, St. Louis, MO, USA), desalted using Oasis^®^ HLB plates (Waters) and vacuum evaporated. Sample study peptides (130 µg) were fractionated using strong cation exchange (SCX) chromatography. Elution was performed using a linear salt gradient and peptides were collected into 8 fractions and freeze dried. SCX fractionation performance was monitored by injecting a peptide mix (Leucine Enkephalin, Oxytocin, Angiotensin I, [Arg8] Vasopressin, [Val4] Angiotensin III, α-Endorphin, in-house preparation) at the beginning and end of each SCX run. Following freeze drying of the SCX fractions, they were resuspended in 1% trifluoroacetic acid and desalted using Oasis^®^ HLB plates. The eluate from the desalting step was split and transferred into two 96-well plates, one plate for LC-MS/MS analysis and the other plate as a back-up. Both plates were vacuum evaporated and stored at −20 °C until analysis by LC-MS/MS.

The SCX fractionated samples (~17 µg per well, 8 study samples × 8 fractions) were resolubilized in 33 µL of 96.25/3.75 (*v*/*v*) water/acetonitrile and 0.2% formic acid, containing five internal standard peptides (FSDISAAK, ASSILAT, NVDQSLLELHK, QNNGAFDETLFR, ELWFSDDPDVTK). These five peptides elute at different retention times throughout the gradient and are used to monitor instrument variability during the LC-MS/MS analysis. Five µL of each sample (~2.5 µg) was injected onto a nanoAcquity UPLC (ultra performance liquid chromatography, Waters) coupled to a Q Exactive mass spectrometer (Thermo Fisher). Survey and tandem mass spectrometry scans were acquired in the same run. Peptide separation was achieved using a nanoAcquity UPLC Trap column Symmetry C18, 180 µm × 20 mm, 5 µm, and a nanoAcquity UPLC column BEH130 C18, 150 µm × 100 mm, 1.7 µm (Waters). The flow rate was 1.8 µL/min.

For protein identification and metaclustering, LC-MS/MS spectra were submitted to a database search using Mascot software v2.2.06 (Matrix Science, Columbia, SC, USA), with search parameters: enzyme = trypsin, allowed missed cleavages = 2, peptide tolerance = 20 ppm, MS/MS tolerance = 0.05 Da, variable modifications = Deamidation (N), Oxidation (M). A custom database was used which included all bacteria from the Human Microbiome Project [54] (downloaded from Uniprot 20140605), plus additional taxonomies identified on the same 8 fecal samples via 16S rRNA gene sequencing (downloaded 20140605), and Uniprot Human (reviewed entries only, downloaded 20140120). A decoy reverse database was used to evaluate the false positive error rate, and Peptide/Protein Teller was used to derive the simplest list of proteins to explain observed peptides with False Discovery Rate = 1.6% at the protein level. Identified proteins were grouped into metaclusters if they shared the same gene name and/or peptide evidence and/or the same Uniref50 identifier [55]. For the subsequent analyses, only organisms/genera/metaclusters/proteins with unique peptide evidence were considered. A peptide was considered unique if its sequence could only be assigned to a single organism, genus, metacluster or protein. The unique nature of a peptide was determined independently at the corresponding level. Conversely, an organism, genus, metacluster or protein were considered identified by a single unique peptide sequence, although the level of confidence in each identification increased with the number of corresponding unique and non-unique peptide assignments.

Protein metaclusters with peptide redundancy removed at the genus level, were submitted to pathway analysis using the ‘KEGG Mapper–Search&Color Pathway’ mapping tool [36,37], and comparative analysis was performed on the different infant categories (e.g., treated with antibiotic, feeding mode, etc.). Pathways with obvious differences between infant categories were evaluated further by extracting the corresponding pathway genes and spectral count data.

### 4.4. Availability of Data and Materials

Raw pyrosequencing data for all samples are available in the Sequence Read Archive under accession number PRJEB19801. The mass spectrometry proteomics data have been deposited into the ProteomeXchange Consortium via the PRIDE [56] partner repository with the dataset identifiers PXD006033 and 10.6019/PXD006033.

### 4.5. Ethics Approval and Consent to Participate

Prior to inclusion in the study, written informed consent was obtained from the parents of all subjects to evaluate fecal microbiota composition and to include relevant subject metadata (age, gender, breast or formula fed, vaginal or C-section delivery, and treatments proximal to sample collection (antibiotics, vaccination)).

The project proposal, including the Informed Consent text and recruitment materials, was submitted to an accredited Medical Research Ethics Committee (Independent Review Board Nijmegen (IRBN)) for confirmation that this project did not need a formal ethical committee review according to Dutch law.

## 5. Conclusions

Metaproteomics data yielded far more information than what was possible from 16S rRNA gene sequencing of the same samples. More organisms were identified with better resolution in each subject. In addition, metabolic pathway information was obtained that can provide a deeper insight into the physiological state of the microbiome, potentially providing diagnostic insights that cannot be derived from DNA sequencing analysis alone. This approach may be particularly important from a diagnostic perspective, given that changes to the proteome may occur more rapidly or independently from taxonomy distribution changes in response to challenges. Protein and pathway changes are also much more readily reconciled with changes to metabolite concentrations, thus opening the door to a better understanding of the impact of the microbiome on the metabolome. For these reasons, fecal metaproteomics is a powerful technology that may improve characterization of the gut microbiome and catalyzes a further understanding of its impact on health and disease outcomes.

## Figures and Tables

**Table 1:** Assignment of spectral counts to unique peptide sequences and metaclusters.

Source	Annotation	#Spectral Counts ^2^	# Unique Peptide Sequences ^3^	# Metaclusters ^4^
Bacterial	With useful annotation ^1^	32,469	10,675	1033
	Without useful annotation	8455	3541	889
Human	With useful annotation	8951	1034	212

^1^ Useful annotation is based on association of a characterized gene name. ^2^ Spectral counts are the number of spectra assigned to a peptide sequence. ^3^ Unique peptide sequences can be assigned to only a single protein group. ^4^ Metaclusters are created by clustering peptides sharing the same gene name and/or peptide evidence and/or the same Uniref50 identifier, independent of species assignment.

**Table 2:** Number of strain–level assignments by metaproteomics in each infant. These numbers are based on assignments of each MS/MS (tandem mass spectrometry) fragmentation spectrum to a peptide sequence and the assignment of that sequence uniquely to a particular bacterial strain.

Subject	Spectral Counts							
	C	T	B	P	M	N	E	R
Total number of strains assigned with ≥1 unique spectrum ^1^	45	67	78	99	75	76	64	64
Number of strains assigned with ≥2 spectra	25	43	49	71	45	42	39	42
Number of strains assigned with ≥5 spectra	9	14	20	32	18	18	19	10

^1^ Level of confidence increases with the number of spectra assigned to the same strain.

**Table 3:** Infant fecal sample characteristics.

Subject ID	C	T	B	P	M	N	E	R
Gestational age (weeks)	39	39	40	41	34	34	40	41
Gender	M	M	M	F	M	M	F	M
Age (days)	64	134	119	61	96	96	140	132
Delivery Mode	V	V	V	CS	V	V	V	V
Feeding Mode	BF	BF	BF	Mixed	Mixed	Mixed	FF	BF
Sample weight (g)	0.23	0.51	0.56	0.55	0.51	0.58	0.52	0.53
Remarks				Vaccinated on sampling day (DTPP/Hib/HepB/Pneu)	Mono-zygotic twin #1	Mono-zygotic twin #2		Antibiotic treatment (amoxicillin)

Gender: M = male, F = female; Delivery mode: V = vaginal, CS = C-section; Feeding Mode: BF = Breastfed, Mixed = breastfed and formula fed, FF = formula fed. Vaccine abbreviations used; DTPP = diphtheria, tetanus, polio and pertussis, Hib = Haemophilus influenza type B, HepB = Hepatitis B, Pneu = Pneumococcal disease.

## Supplementary Materials

Supplementary materials can be found at https://www.mdpi.com/1422-0067/20/6/1430/s1. Supplementary Table S1: Overall proteomics results; Supplementary Table S2: Proteomics list of organisms; Supplementary Table S3: Pyrosequencing list of organisms; Supplementary Table S4: Identified strains.

## Abbreviations

C-section	Caesarean-section
LC-MS/MS	liquid chromatography-tandem mass spectrometry
TCA	tricarboxylic acid
HMO	human milk oligosaccharides
SCXUPLC	strong cation exchangeultra performance liquid chromatography

## References

[1] Kostic A.D., Gevers D., Siljander H., Vatanen T., Hyotylainen T., Hamalainen A.M., Peet A., Tillmann V., Poho P., Mattila I. (2015). The dynamics of the human infant gut microbiome in development and in progression toward type 1 diabetes. Cell Host Microbe.

[2] Wopereis H., Oozeer R., Knipping K., Belzer C., Knol J. (2014). The first thousand days—Intestinal microbiology of early life: Establishing a symbiosis. Pediatr. Allergy Immunol..

[3] Bokulich N.A., Chung J., Battaglia T., Henderson N., Jay M., Li H., DLieber A., Wu F., Perez-Perez G.I., Chen Y. (2016). Antibiotics, birth mode, and diet shape microbiome maturation during early life. Sci. Transl. Med..

[4] Yassour M., Vatanen T., Siljander H., Hämäläinen A.-M., Härkönen T., Ryhänen S.J., Franzosa E.A., Vlamakis H., Huttenhower C., Gevers D. (2016). Natural history of the infant gut microbiome and impact of antibiotic treatment on bacterial strain diversity and stability. Sci. Transl. Med..

[5] Korpela K., Salonen A., Virta L.J., Kekkonen R.A., de Vos W.M. (2016). Association of Early-Life Antibiotic Use and Protective Effects of Breastfeeding: Role of the Intestinal Microbiota. JAMA Pediatr..

[6] Langdon A., Crook N., Dantas G. (2016). The effects of antibiotics on the microbiome throughout development and alternative approaches for therapeutic modulation. Genome Med..

[7] Azad M.B., Konya T., Maughan H., Guttman D.S., Field C.J., Chari R.S., Sears M.R., Becker A.B., Scott J.A., Kozyrskyj A.L. (2013). Gut microbiota of healthy Canadian infants: Profiles by mode of delivery and infant diet at 4 months. Can. Med. Assoc. J..

[8] Chen J., Cai W., Feng Y. (2007). Development of intestinal bifidobacteria and lactobacilli in breast-fed neonates. Clin. Nutr..

[9] Jost T., Lacroix C., Braegger C.P., Chassard C. (2012). New Insights in Gut Microbiota Establishment in Healthy Breast Fed Neonates. PLoS ONE.

[10] Roger L.C., Costabile A., Holland D.T., Hoyles L., McCartney A.L. (2010). Examination of faecal Bifidobacterium populations in breast- and formula-fed infants during the first 18 months of life. Microbiology.

[11] Martin R., Makino H., Cetinyurek Yavuz A., Ben-Amor K., Roelofs M., Ishikawa E., Kubota H., Swinkels S., Sakai T., Oishi K. (2016). Early-Life Events, Including Mode of Delivery and Type of Feeding, Siblings and Gender, Shape the Developing Gut Microbiota. PLoS ONE.

[12] Bäckhed F., Roswall J., Peng Y., Feng Q., Jia H., Kovatcheva-Datchary P., Li Y., Xia Y., Xie H., Zhong H. (2015). Dynamics and Stabilization of the Human Gut Microbiome during the First Year of Life. Cell Host Microbe.

[13] Putignani L., Del Chierico F., Petrucca A., Vernocchi P., Dallapiccola B. (2014). The human gut microbiota: A dynamic interplay with the host from birth to senescence settled during childhood. Pediatr. Res..

[14] Dominguez-Bello M.G., Costello E.K., Contreras M., Magris M., Hidalgo G., Fierer N., Knight R. (2010). Delivery mode shapes the acquisition and structure of the initial microbiota across multiple body habitats in newborns. Proc. Natl. Acad. Sci. USA.

[15] Bergström A., Skov T.H., Bahl M.I., Roager H.M., Christensen L.B., Ejlerskov K.T., Mølgaard C., Michaelsen K.F., Licht T.R. (2014). Establishment of Intestinal Microbiota during Early Life: A Longitudinal, Explorative Study of a Large Cohort of Danish Infants. Appl. Environ. Microbiol..

[16] Huda M.N., Lewis Z., Kalanetra K.M., Rashid M., Ahmad S.M. (2014). Stool Microbiota and Vaccine Responses of Infants. Pediatrics.

[17] Kim M., Oh H.-S., Park S.-C., Chun J. (2014). Towards a taxonomic coherence between average nucleotide identity and 16S rRNA gene sequence similarity for species demarcation of prokaryotes. Int. J. System. Evol. Microbiol..

[18] Sim K., Cox M.J., Wopereis H., Martin R., Knol J., Li M.-S., Cookson W.O.C.M., Moffatt M.F., Kroll J.S. (2012). Improved Detection of Bifidobacteria with Optimised 16S rRNA-Gene Based Pyrosequencing. PLoS ONE.

[19] Case R.J., Boucher Y., Dahllöf I., Holmström C., Doolittle W.F., Kjelleberg S. (2007). Use of 16S rRNA and rpoB Genes as Molecular Markers for Microbial Ecology Studies. Appl. Environ. Microbiol..

[20] Stackebrandt E., Goebel B.M. (1994). Taxonomic Note: A Place for DNA-DNA Reassociation and 16S rRNA Sequence Analysis in the Present Species Definition in Bacteriology. Int. J. System. Evol. Microbiol..

[21] Qin J., Li R., Raes J., Arumugam M., Burgdorf K.S., Manichanh C., Nielsen T., Pons N., Levenez F., Yamada T. (2010). A human gut microbial gene catalogue established by metagenomic sequencing. Nature.

[22] Land M., Hauser L., Jun S.-R., Nookaew I., Leuze M.R., Ahn T.-H., Karpinets T., Lund O., Kora G., Wassenaar T. (2015). Insights from 20 years of bacterial genome sequencing. Function. Integr. Genom..

[23] Jones M.B., Highlander S.K., Anderson E.L., Li W., Dayrit M., Klitgord N., Fabani M.M., Seguritan V., Green J., Pride D.T. (2015). Library preparation methodology can influence genomic and functional predictions in human microbiome research. Proc. Natl. Acad. Sci. USA.

[24] Sinha R., Abnet C.C., White O., Knight R., Huttenhower C. (2015). The microbiome quality control project: Baseline study design and future directions. Genome Biol..

[25] Wilmes P., Heintz-Buschart A., Bond P. (2015). A decade of metaproteomics: Where we stand and what the future hold. Proteomics.

[26] Xiong W., Abraham P.E., Li Z., Pan C., Hettich R.L. (2015). Microbial metaproteomics for characterizing the range of metabolic functions and activities of human gut microbiota. Proteomics.

[27] Kolmeder C.A., de Vos W.M. (2014). Metaproteomics of our microbiome -developing insight in function and activity in man and model systems. J. Proteom..

[28] Abraham P., Adams R., Giannone R.J., Kalluri URanjan P., Erickson B., Shah M., Tuskan G.A., Hettich R.L. (2011). Defining the boundaries and characterizing the landscape of functional genome expression in vascular tissues of Populus using shotgun proteomics. J. Proteome Res..

[29] VerBerkmoes N.C., Denef V.J., Hettich R.L., Banfield J.F. (2009). Systems biology: Functional analysis of natural microbial consortia using community proteomics. Nat. Rev. Microbiol..

[30] Zhang X., Chen W., Ning Z., Mayne J., Mack D., Stintzi A., Tian R., Figeys D. (2017). Deep Metaproteomics Approach for the Study of Human Microbiomes. Anal. Chem..

[31] Young J.C., Pan C., Adams R., Brooks B., Banfield J.F., Morowitz M.J., Hettich R.L. (2015). Metaproteomics Reveals Functional Shifts in Microbial and Human Proteins During a Preterm Infant Gut Colonization Case. Proteomics.

[32] Klaassens E.S., de Vos W.M., Vaughan E.E. (2007). Metaproteomics Approach to Study the Functionality of the Microbiota in the Human Infant Gastrointestinal Tract. Appl. Environ. Microbiol..

[33] Xiong W., Giannone R.J., Morowitz M.J., Banfield J.F., Hettich R.L. (2015). Development of an enhanced metaproteomic approach for deepening the microbiome characterization of the human infant gut. J. Proteome Res..

[34] Xiong W., Brown C.T., Morowitz M.J., Banfield J.F., Hettich R.L. (2017). Genome-resolved metaproteomic characterization of preterm infant gut microbiota development reveals species-specific metabolic shifts and variabilities during early life. Microbiome.

[35] Zwittink R.D., van Zoeren-Grobben D., Martin R., van Lingen R.A., Groot Jebbink L.J., Boeren S., Renes I.B., van Elburg R.M., Belzer C., Knol J. (2017). Metaproteomics reveals functional differences in intestinal microbiota development of preterm infants. Mol. Cell Proteom..

[36] Kanehisa M., Goto S. (2000). KEGG: Kyoto Encyclopedia of Genes and Genomes. Nucleic Acids Res..

[37] Kanehisa M., Sato Y., Kawashima M., Furumichi M., Tanabe M. (2016). KEGG as a reference resource for gene and protein annotation. Nucleic Acids Res..

[38] Ruiz-Moyano S., Totten S.M., Garrido D.A., Smilowitz J.T., German J.B., Lebrilla C.B., Mills D.A. (2013). Variation in Consumption of Human Milk Oligosaccharides by Infant Gut-Associated Strains of Bifidobacterium breve. Appl. Environ. Microbiol..

[39] Marcobal A., Barboza M., Sonnenburg E.D., Pudlo N., Martens E.C., Desai P., Lebrilla C.B., Weimer B.C., Mills D.A., German J.B. (2011). Bacteroides in the Infant Gut Consume Milk Oligosaccharides via Mucus-Utilization Pathways. Cell Host Microbe.

[40] Francino M.P. (2016). Antibiotics and the Human Gut Microbiome: Dysbioses and Accumulation of Resistances. Front Microbiol..

[41] Junick J., Blaut M. (2012). Quantification of human fecal bifidobacterium species by use of quantitative real-time PCR analysis targeting the groEL gene. Appl. Environ. Microbiol..

[42] Guaraldi F., Salvatori G. (2012). Effect of Breast and Formula Feeding on Gut Microbiota Shaping in Newborns. Front. Cell. Infect. Microbiol..

[43] Underwood M.A., German J.B., Lebrilla C.B., Mills D.A. (2015). Bifidobacterium longum subspecies infantis: Champion colonizer of the infant gut. Pediatr. Res..

[44] James K., O’Connell Motherway M., Bottacini F., van Sinderen D. (2016). Bifidobacterium breve UCC2003 metabolises the human milk oligosaccharides lacto-*N*-tetraose and lacto-*N*-neo-tetraose through overlapping, yet distinct pathways. Sci. Rep..

[45] Goodrich Julia K., Davenport Emily R., Beaumont M., Jackson Matthew A., Knight R., Ober C., Spector Tim D., Bell Jordana T., Clark Andrew G., Ley Ruth E. (2016). Genetic Determinants of the Gut Microbiome in UK Twins. Cell Host Microbe.

[46] Heine W., Mohr C., Wutzke K.D. (1992). Host-microflora correlations in infant nutrition. Prog. Food Nutr. Sci..

[47] Pible O., Armengaud J. (2015). Improving the quality of genome, protein sequence, and taxonomy databases: A prerequisite for microbiome meta-omics 2.0. Proteomics.

[48] Tanca A., Palomba A., Fraumene C., Pagnozzi D., Manghina V., Deligios M., Muth T., Rapp E., Martens L., Addis M.F. (2016). The impact of sequence database choice on metaproteomic results in gut microbiota studies. Microbiome.

[49] Matsuki T., Watanabe K., Fujimoto J., Takada T., Tanaka R. (2004). Use of 16S rRNA Gene-Targeted Group-Specific Primers for Real-Time PCR Analysis of Predominant Bacteria in Human Feces. Appl. Environ. Microbiol..

[50] Caporaso J.G., Kuczynski J., Stombaugh J., Bittinger K., Bushman F.D., Costello E.K., Fierer N., Peña A.G., Goodrich J.K., Gordon J.I. (2010). QIIME allows analysis of high-throughput community sequencing data. Nat. Methods.

[51] Pruesse E., Quast C., Knittel K., Fuchs B.M., Ludwig W., Peplies J., Glöckner F.O. (2007). SILVA: A comprehensive online resource for quality checked and aligned ribosomal RNA sequence data compatible with ARB. Nucleic Acids Res..

[52] Edgar R.C. (2010). Search and clustering orders of magnitude faster than BLAST. Bioinformatics.

[53] Edgar R.C. (2013). UPARSE: Highly accurate OTU sequences from microbial amplicon reads. Nat. Meth..

[54] Turnbaugh P.J., Ley R.E., Hamady M., Fraser-Liggett C.M., Knight R., Gordon J.I. (2007). The Human Microbiome Project. Nature.

[55] Suzek B.E., Wang Y., Huang H., McGarvey P.B., Wu C.H., The UniProt Consortium (2015). UniRef clusters: A comprehensive and scalable alternative for improving sequence similarity searches. Bioinformatics.

[56] Vizcaíno J.A., Csordas A., del-Toro N., Dianes J.A., Griss J., Lavidas I., Mayer G., Perez-Riverol Y., Reisinger F., Ternent T. (2016). 2016 update of the PRIDE database and related tools. Nucleic Acids Res..

